# Spontaneous rectus sheath hematoma in pregnancy and a systematic anatomical workup of rectus sheath hematoma: a case report

**DOI:** 10.1186/s13256-016-1081-6

**Published:** 2016-10-19

**Authors:** Kerstin Eckhoff, Thilo Wedel, Marcus Both, Kayhan Bas, Nicolai Maass, Ibrahim Alkatout

**Affiliations:** 1 Department of Gynecology and Obstetrics, Kiel School of Gynaecological Endoscopy, University Hospitals Schleswig-Holstein, Campus Kiel, Arnold-Heller Str. 3, House 24, 24105 Kiel, Germany; 2 Institute of Anatomy, Christian-Albrechts-University in Kiel, Olshausenstraße 8, 24105 Kiel, Germany; 3 Department of Radiology, University Hospitals Schleswig-Holstein, Campus Kiel, Arnold-Heller Str. 3, House 41, 24105 Kiel, Germany; 4 Department of General Surgery, University Hospitals Schleswig-Holstein, Campus Kiel, Arnold-Heller Str. 3, House 18, 24105 Kiel, Germany

**Keywords:** Rectus sheath hematoma, Pregnancy, Case report, Abdominal pain, Anatomy abdominal wall

## Abstract

**Background:**

Rectus sheath hematoma is a rare clinical diagnosis, particularly in pregnancy. Due to unspecific symptoms, misdiagnosis is likely and could potentially endanger a patient as well as her fetus.

**Case presentation:**

A 26-year-old white woman presented with mild right-sided abdominal pain, which increased during palpation and movement, at 26 + 3 weeks’ gestational age. Ultrasound imaging initially showed a round and well-demarcated structure, which appeared to be in contact with her uterine wall, leading to a suspected diagnosis of an infarcted leiomyoma. However, she reported increasing levels of pain and laboratory tests showed a significant drop in her initially normal hemoglobin level. A magnetic resonance imaging scan finally revealed a large type III rectus sheath hematoma on the right side. Because of progressive blood loss into her rectus sheath under conservative therapy, with a significant further decrease in her hemoglobin levels, surgical treatment via right-sided paramedian laparotomy was initiated. During the operation the arterial bleed could be ligated. She eventually achieved complete convalescence and delivered a healthy newborn spontaneously after 40 weeks of gestation.

**Conclusion:**

This case report highlights the clinical and diagnostic features of rectus sheath hematoma and shows the anatomical aspects of the rectus sheath, simplifying early and correct diagnosis.

## Background

Rectus sheath hematoma (RSH) is a rare condition and only a few cases during pregnancy have been reported [1]. RSH results from a rupture of one of the epigastric arteries leading to an acute bleeding into the rectus sheath. Due to anatomical conditions these hematomas may contain high amounts of blood, thus leading to hypovolemia and hemorrhagic shock and possible maternal and fetal mortality. Particularly in pregnancy, high mortality rates of up to 13 % for the mother and 50 % for the fetus have been reported [2].

In many cases diagnosis remains difficult as the symptoms of RSH may mimic several other clinical conditions. Tolcher *et al*. reviewed ten cases of spontaneous RSH in pregnancy. In 50 % of these cases, immediate caesarean section was performed, eventually leading to preterm deliveries [1]. In all of these prescribed cases, a correct preoperative diagnosis was missing, indicating its high importance. To facilitate diagnosis, we analyzed the clinical and radiological features of RSH and highlighted the pathophysiology via a systematic anatomic workup.

## Case presentation

A 26-year-old white woman (II gravida/I para) presented at the gestational age of 26 + 3 weeks with right-sided abdominal pain. The course of her pregnancy up to this point was normal. There had been no pre-existing illnesses. She merely reported a flu-like infection associated with severe coughing fits the week before, which was almost in complete remission. She had not received any anticoagulant therapy and had no indications for any other anticoagulant abnormalities. No trauma could be recollected and there had been no further surgical interventions.

**Fig. 1:**
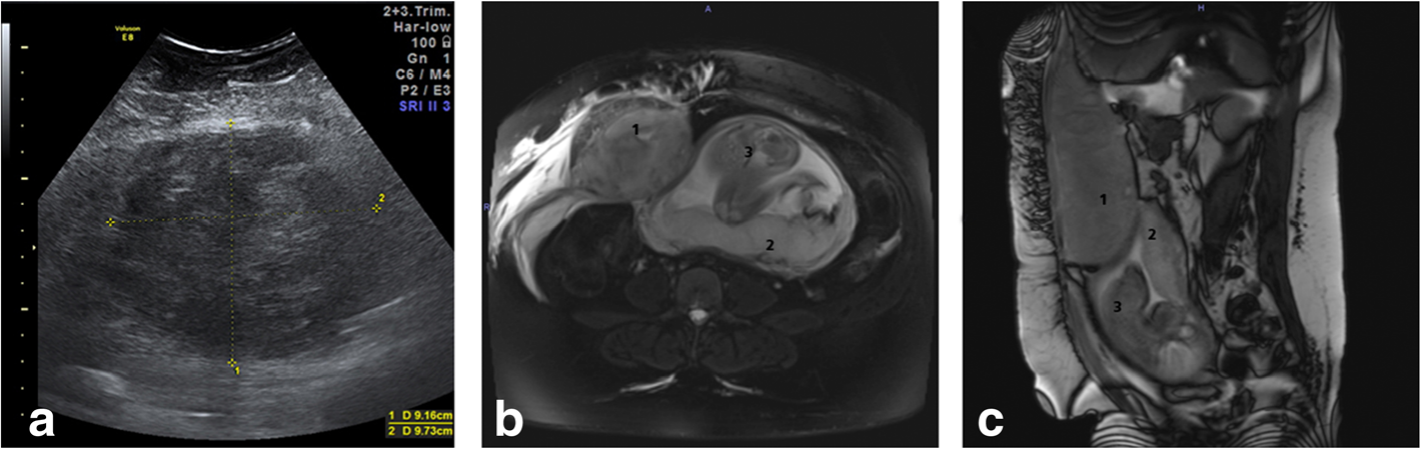
**a** A 26-year-old pregnant woman at 27 weeks’ gestation presented with right-sided abdominal pain. Transversal transabdominal ultrasound of her right lumbar region showed a 9.16 × 9.73 cm, round, well-demarcated and partly hypoechoic/isoechoic structure, appearing to be in contact with her uterine wall. **b** Magnetic resonance imaging of her abdomen, coronal view: Type III rectus sheath hematoma (*1*) measuring 11 × 12 × 20 cm, mainly located at the right side and starting to displace her uterine wall. The placenta (*2*) is located at the rear aspect of her uterus and appears to be normal, without sign of acute abruptio placentae. **c** Magnetic resonance imaging of her abdomen, axial view: The fetus (*3*) is lying in cephalic presentation. Note the associated edema of the subcutaneous tissue

On admission, she presented hemodynamically stable with vital signs within normal limits. Physical and pelvic examinations appeared normal apart from moderate maternal obesity and a mild tenderness in her right upper quadrant which increased when moving. Ultrasound revealed a normal progression of pregnancy, regular percentiles of fetal growth, and a normal Doppler ultrasound state. The placenta was located at the rear aspect of her uterus without indications for a retroplacental hematoma or acute abruptio placentae. However, a 9.16 × 9.73 cm, round, well-demarcated, and partly hypoechoic/isoechoic structure, which appeared to be in contact with her uterine wall but clearly separated from the placenta, was identified (Fig. 1a). Its inhomogeneous internal structure led to the suspected diagnosis of an acute symptomatic infarcted pedunculated myoma. As a possible differential diagnosis we considered a hematoma of unknown origin. However, initial laboratory findings were consistent with our first hypothesis and did not show a typical HELLP (hemolysis, elevated liver enzymes, low platelet count) constellation or other severe abnormalities. Her hemoglobin (Hb) level was 11.8 g/dl and her level of C-reactive protein (CRP) was slightly elevated at 20.4 mg/l. Furthermore, her coagulation parameters appeared to be normal.

We initiated analgesic therapy with acetaminophen and piritramide as well as intramuscular dexamethasone injection as a fetal respiratory distress prophylaxis.

Initially, her abdominal pain subsided mildly and cardiotocography (CTG) monitoring showed physiological values. On day 2 after admission, she reported an acute exacerbation of her abdominal pain. Sonographic reevaluation indicated an increase in size of the aforementioned structure. Laboratory findings showed a significant decrease in Hb to 8.1 g/dl, negating our initial diagnosis. We suspected an acute hematoma of unknown origin or an abdominal bleed and initiated an emergency magnetic resonance imaging (MRI) scan, which showed a RSH measuring 11 × 12 × 20 cm that was located at the right side of her anterior abdominal wall (Fig. 1b, c).

**Fig. 2:**
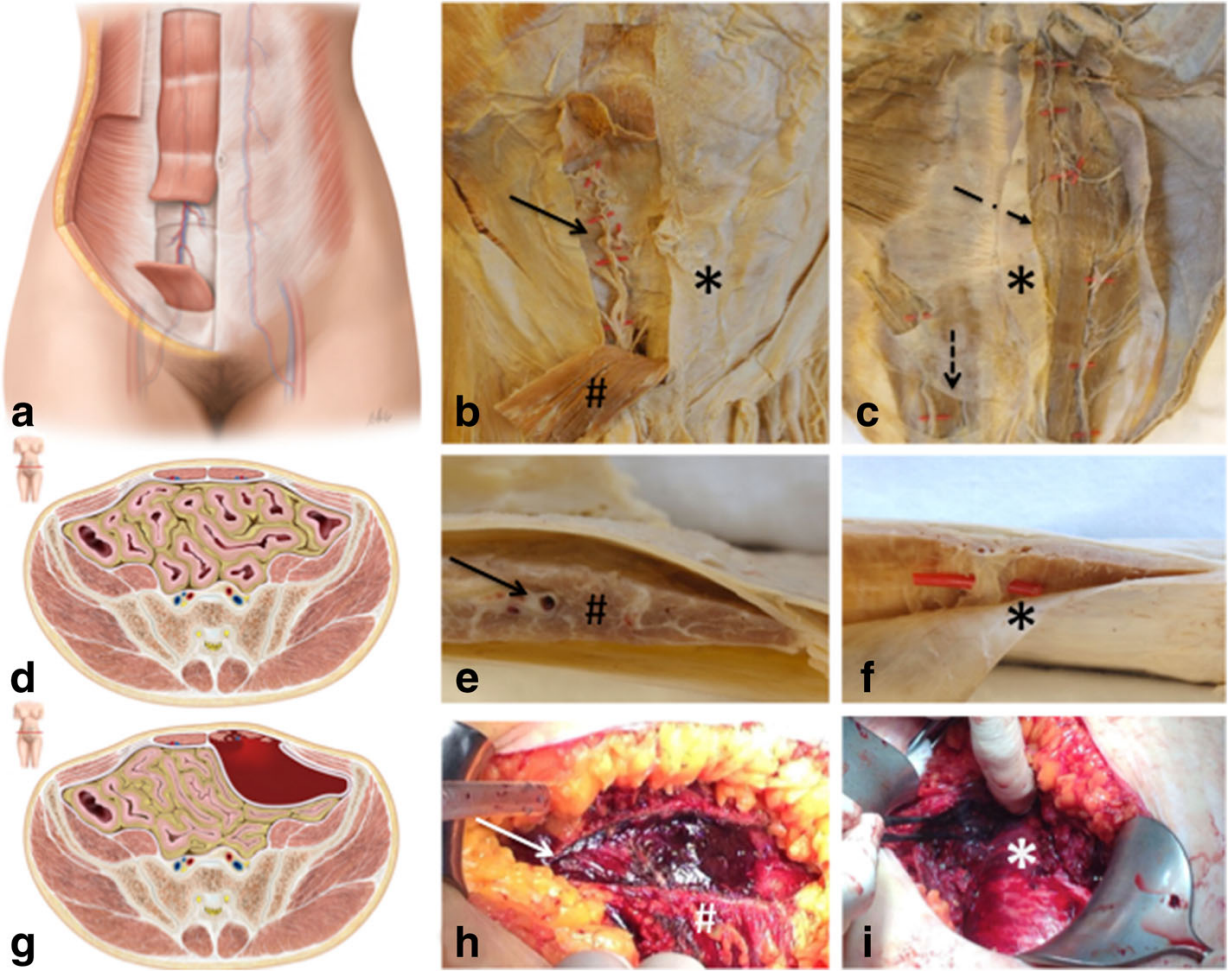
**a** Virtualization of the anterior abdominal wall: On the left side the oblique external muscle is shown, located on top of the three flat muscles of the lateral abdominal wall. On the right-hand side multiple layers were excluded to show the underlying internal oblique muscle and the transversus abdominis. The rectus sheath forms a central part of the functional system. It contains the rectus abdominis and the pyramidalis muscle. **b** Cadaver study, abdominal wall, ventral view: opened rectus sheath (*); clearly visible is the rectus abdominis muscle (#) with its intramuscular intersections. *Red marks* and arrow highlight the underlying inferior epigastric artery. **c** Cadaver study, abdominal wall, dorsal view showing the linea alba, running longitudinally. After removing the aponeurosis of the transversus abdominis muscle (*), the underlying inferior epigastric artery and the rectus abdominis muscle are visible. Arterial branches are mainly located behind intramuscular intersections. The second arrow is pointing to the intramuscular intersections (*oblique arrow*). The *perpendicular arrow* marks the linea arcuata. **d** Virtualization of the normal anatomy, cranial aspect of the linea arcuata, axial view: The ventral and dorsal limitations of the rectus sheath differ between the cranial and caudal aspect of the structure. Superior to the arcuate line, the layers of the aponeurosis of the transversus abdominis muscle are separated and run anteriorly and posteriorly of the musculus rectus abdominis. Inferior to the linea arcuata, the aponeuroses of all three muscles run anteriorly and the rectus sheath is separated from the peritoneum only by the transversalis fascia. **e** Cadaver study, cross section, axial view showing the rectus abdominis muscle (#). The inferior epigastric artery runs longitudinally within the rectus sheath. The rectus muscle (#) and the rectus sheath. The arrow is pointing to the centrally localized epigastric artery. **f** Cadaver study, cross section, axial view: *Red marks* highlight the inferior epigastric artery, a branch of the external iliac artery and the rectus sheath (*). **g** Virtualization of a rectus sheath hematoma, cranial aspect of the linea arcuata, axial view: Rupture of the rectus abdominis muscle with a significant arterial bleed of the inferior epigastric artery and a subsequent hematoma, typically not surpassing the ipsilateral aspect of the linea alba. **h** Operative finding: After incision of the lamina anterior fasciae rectus abdominis the partly consolidated hematoma (*white arrow*) was recovered, measuring approximately 1000 ml of fresh and coagulated blood. The cranial aspect of the rectus abdominis muscle (#) was severed and multiple small intramuscular hematomas were noted. At the caudal aspect a sputtering hemorrhage of the inferior epigastric artery was found and both ends were ligated. **i** Operative finding: The fascia of the transversus abdominis (*) muscle remained intact. No caesarean section was performed

We started conservative treatment with analgesics and balanced intravenous fluids. After a few hours she developed severe and progressive pain. Repetitive laboratory tests showed a further decrease in Hb levels. She developed hypotension, indicating surgical exploration by right-sided paramedian laparotomy. After incision of the anterior fasciae of her rectus sheath the hematoma was recovered, measuring approximately 1000 ml of fresh and coagulated blood. At the caudal aspect a sputtering hemorrhage of the inferior epigastric artery was found and both ends were ligated (Fig. 2). Two 16G drains were placed and put under suction. A total of 4 units of packed red blood cells were transfused. Intraoperative fetal monitoring was conducted by transabdominal ultrasound and showed normal fetal heart rates. After the procedure her Hb levels remained stable and her complete convalescence was swift. She was discharged on day 5 post surgery. Fetal parameters appeared to be normal.

At 40 weeks’ of gestational age she underwent normal spontaneous vaginal delivery under intensified peripartum controls (Table 1).

**Table 1:** Timeline of case

Admission to hospital	29 January 2016
Operation procedure	30 January 2016
Discharge from hospital	04 February 2016
Spontaneous vaginal delivery	04 May 2016

## Discussion

### Systematic anatomical workup and pathophysiology of a RSH

#### Anatomy of the rectus sheath

To depict the relevant anatomical structures for the pathogenesis of RSH, we performed a cadaver study using formalin-fixed bodies of cadaver donors.

The rectus sheath forms a central part of the anterior abdominal wall. It connects the xiphoid process and the costal cartilages of the fifth, sixth and the seventh rib with the os pubis and contains two muscles: the rectus abdominis and the pyramidalis muscle. The medial border is formed by the fibrous linea alba and the lateral border is formed by aponeuroses of the external oblique, the internal oblique, and the transversus abdominis, building the linea semilunaris. The ventral and dorsal limitations differ between the cranial and caudal aspect of the structure. The linea arcuata runs horizontally and is located approximately 5 cm caudal to the umbilicus. Inferior to the linea arcuata, the aponeuroses of all three muscles run anteriorly and the rectus sheath is separated from the peritoneum only by the transversalis fascia. Superior to the arcuate line, the layers of the aponeurosis of the transversus abdominis muscle are separated and run anteriorly and posteriorly of the musculus rectus abdominis (Fig. 2c) [3–5].

The blood supply is provided by the epigastric arteries, which run longitudinally within the rectus sheath (Fig. 2d–f) [6, 7].

#### Pathophysiology and risk factors

RSHs are mostly caused by trauma of the inferior epigastric artery. Whetzel and Huang [8] showed a higher number of branches originating from the inferior epigastric artery. During contraction of the rectus abdominis muscle these branching sites are burdened and exposed to shearing and straining stresses. Because of this, strong muscular contraction may cause trauma of the inferior epigastric artery, leading to a RSH [8].

Risk factors include conditions that elevate intraabdominal pressure, such as coughing fits, severe vomiting, or straining during passing a stool, but also trauma, hereditary or iatrogenous coagulation abnormalities, vascular malformation, female sex, and previous surgery [9].

### Clinical features

#### Symptoms

Symptoms of profuse bleeding into the rectus sheath are often relatively unspecific and may include sudden or increasing – often unilateral – abdominal pain, palpable masses in the abdominal wall, and general conditions of a hypovolemic state, such as tachycardia, hypotension, tachypnea, and pallor. A clinical examination may show Fothergill’s sign (an abdominal mass which stays palpable and becomes tenderer during contraction of the rectus muscle), Carnett’s sign (an increase of pain on tensing of the abdominal muscles), Cullen’s sign (periumbilical ecchymosis), or Turner’s sign (flank ecchymosis). Furthermore, low-grade fever, nausea, and vomiting have been reported in patients with acute RSHs [10–13].

#### Differential diagnoses

The risk of misinterpreting the aforementioned symptoms is elevated during pregnancy and correct diagnosis may be difficult. Differential obstetric diagnoses include acute abruptio placentae, infarcted myoma, preterm labor, uterine rupture, or HELLP syndrome. There are also multiple non-pregnancy-related differential diseases, such as gastroenteritis, nephritis, nephrolithiasis, perforated ulcer, pancreatic disease, hepatitis, gallbladder disease, acute appendicitis, ovarian torsion, or an intraabdominal bleed [12, 14, 15].

#### Diagnostics

Ultrasound poses a viable diagnostic feature in RSH. It can be used as a first-line test and is also secure in pregnant patients. RSHs, located above the arcuate line, often occur as a unilateral spindle-shaped mass. Underneath the arcuate line hematomas commonly appear spherical [15, 16]. Laboratory results typically show a significant decrease in the Hb level. Furthermore, coagulation abnormalities may be detected.

In non-pregnant patients, a computed tomography (CT) scan is the diagnostic gold standard as it enables a sensitivity and specificity of 100 %. Berna *et al*. found a diagnostic classification of RSH in CT scans [17]. According to this classification, type I hematomas are located intramuscularly and unilaterally. Typically, type II hematomas show blood between the rectus abdominis muscle and the underlying fascia. The more severe type III hematomas show blood in the inner abdomen and the prevesical space [17]. To avoid radiation, MRI scans are a secure alternative in pregnant patients although they may have a lower diagnostic value in acute hematomas within the first 48 hours [18, 19].

#### Treatment

Conservative treatment, including analgesic therapy and fluid resuscitation, is usually possible in hemodynamically stable patients with smaller RSHs. These hematomas will normally resolve themselves within a few weeks [20, 21]. Patients under anticoagulant therapy may additionally benefit from phytonadione or substitution of fresh frozen plasma. In the case of larger hematomas, a transfusion of packed red blood cells is often needed. Hemodynamically unstable patients with expanding hematomas under conservative therapy should undergo invasive treatment, including arterial embolization or surgical decompression. Arterial embolization can be conducted by thrombin or via coiling of the vessel [22]. Classic surgical treatment includes ligation of the arterial bleed and removal of the hematoma. Tolcher *et al*. reported 7 out of 10 cases (70 %) of antepartum RSH in which surgical evacuation was performed [1]. In our reported case, paramedian laparotomy was conducted and finally stopped the bleeding.

### General discussion

RSH is a serious and potentially life-threatening condition, which presents with acute abdominal pain. As in the present report, unspecific symptoms of an acute RSH may mimic several other acute conditions and can easily lead to a wrong diagnosis. To prevent a hemorrhage, which potentially endangers mother and fetus or leads to preterm labor, early diagnosis is important [1].

Diagnosis may be reached by transabdominal ultrasound; however, correct interpretation and location of the multiple compartments of the abdominal wall, especially in pregnant women, may be difficult [15, 19]. Although a CT scan is the gold standard in acute hematomas, a MRI scan poses a viable diagnostic alternative and should always be considered in combination with ultrasound in pregnant patients.

**Fig. 3:**
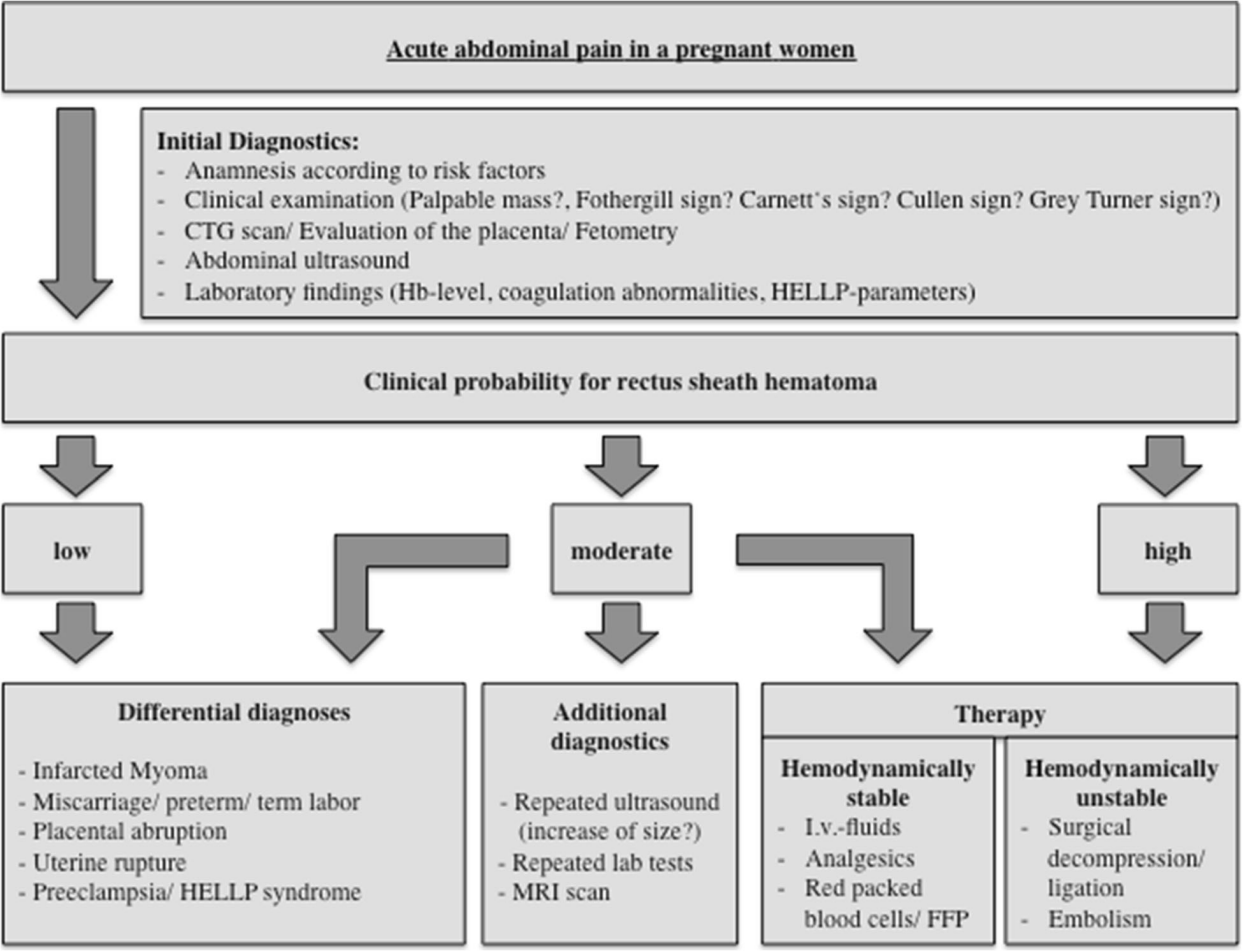
Flow chart for acute abdominal pain in a pregnant woman. Rectus sheath hematoma: clinical features, diagnostics and therapy. *CTG* cardiotocography, *FFP* fresh frozen plasma, *Hb* hemoglobin, *HELLP* hemolysis, elevated liver enzymes, low platelet count, *I.v.* intravenous

After diagnosis, a conservative therapy should be conducted. If the patient presents with hemodynamic instability and a profound bleeding is detected, invasive therapy is indispensable and reduces mortality and morbidity for the mother and the fetus [20] (Fig. 3).

Several cases in the literature report uneventful, spontaneous vaginal delivery after RSH [22, 23]. Although a previous RSH in a patient’s medical history increases her risk of recurrence, spontaneous vaginal delivery seems to be safe and justifiable under intensified intrapartum and postpartum controls. According to this and with our patient’s consent, we planned a spontaneous vaginal delivery which proved uneventful.

## Conclusions

We present a case of a woman with RSH during pregnancy, which initially appeared as a pedunculated myoma. Our cadaver study highlights the anatomical features of the anterior abdominal wall, explaining the pathophysiology of this disease. In conclusion, RSH is a rare diagnosis during pregnancy but should always be considered a potential differential diagnosis of abdominal pain. In case of unspecific symptoms early imaging (for example, via MRI scan) should be taken into consideration as an early and correct diagnosis significantly reduces maternal as well as fetal morbidity and mortality.
